# Registration for public drug benefits across areas of differing ethnic composition in British Columbia, Canada

**DOI:** 10.1186/1472-6963-10-171

**Published:** 2010-06-17

**Authors:** Vivian W Leong, Steve Morgan, Sabrina T Wong, Gillian E Hanley, Charlyn Black

**Affiliations:** 1 Centre for Health Services and Policy Research, University of British Columbia, Vancouver, Canada; 2 School of Population & Public Health, University of British Columbia, Vancouver, Canada; 3 School of Nursing, University of British Columba, Vancouver, Canada

## Abstract

**Background:**

In 2003, the government of British Columbia, Canada introduced a universal drug benefit plan to cover drug costs that are high relative to household income. Residents were required to register in order to be eligible for the income-based benefits. Given past research suggesting that registration processes may pose an access barrier to certain subpopulations, we aimed to determine whether registration rates varied across small geographic areas that differed in ethnic composition.

**Methods:**

Using linked population-based administrative databases and census data, we conducted multivariate logistic regression analyses to determine whether the probability of registration for the public drug plan varied across areas of differing ethnic composition, controlling for household-level predisposing, enabling and needs factors.

**Results:**

The adjusted odds of registration did not differ across regions characterized by high concentrations (greater than 30%) of residents identifying as North American, British, French or other European. Households located in areas with concentrations of residents identifying as an Asian ethnicity had the highest odds of program registration: Chinese (OR = 1.21, CI: 1.19-1.23) and South Asian (OR = 1.19, CI: 1.16-1.22). Despite this positive finding, households residing in areas with relatively high concentrations of recent immigrants had slightly lower adjusted odds of registering for the program (OR = 0.97, CI: 0.95-0.98).

**Conclusions:**

This study identified ethnic variation in registration for a new public drug benefit program in British Columbia. However, unlike previous studies, the variation observed did not indicate that areas with high concentrations of certain ethnicities experienced disadvantages. Potential explanations are discussed.

## Background

### Introduction

The steadily increasing role of prescription drugs in health care systems has made access to medicines a major determinant of health care quality and equity. The availability and extent of prescription drug insurance can influence the use of medicines and thereby affect health outcomes and health services utilization [1,2]. Specifically, financial barriers to accessing medicines are associated with decreases in the use of essential (e.g. life sustaining medicines and medicines important for the treatment of chronic conditions) and non-essential medicines; moreover decreases in appropriate medicine use are associated with increases in health care utilization (e.g. emergency room use) [1,2]. In order to prevent health disparities arising from inequities in access to medicines, a variety of public drug benefit programs have evolved in the USA and Canada to make medicines more accessible and affordable for certain populations. Some of these programs require that potentially eligible beneficiaries register to take part. Past research suggests that registration processes for public health insurance programs can pose an access barrier for certain subpopulations, including those defined by ethnic characteristics [3,4]. The primary goal of our study was to examine whether the probability of registration for a new public drug benefit program in British Columbia, Canada varied across areas of differing ethnic composition.

### Background

The Canadian health care system is publically funded, providing physician and hospital services to all Canadians and residents [5]. The responsibility of providing health care services is delegated to provincial governments, who must follow national guidelines to uphold principles such as equity and accessibility. In contrast to the provision of health care services, publicly funded drug benefit programs are not nationally regulated in Canada. Consequently, a variety of programs with different levels of coverage and eligibility criteria have emerged across provinces.

Historically, provincial drug benefit programs have operated as an age-entitlement: residents not on welfare typically received little or no public subsidy until they turned 65, after which they would automatically qualify for relatively comprehensive public drug benefits [6]. However, motivated by equity considerations regarding access to medicines and related financial burdens for non-senior (aged ≤ 64) populations, a growing number of provinces are implementing income-based benefit programs that provide public subsidies for any resident (regardless of age) whose prescription drug costs are high relative to their incomes. The province of British Columbia (BC) did so in 2003 by introducing what it called "Fair PharmaCare" [7].

The Fair PharmaCare program is a publicly-funded drug insurance program under which annual deductibles and co-insurance rates are set - on a 'sliding scale' - according to household income. The program is universal in the sense that all residents are eligible for benefits; however the fact that the terms of coverage are explicitly income-based means that persons who wish to be covered must register for the program and consent to have their household income verified annually with the Canada Revenue Agency. Based on previous research [4,8,9], we conjecture that the requirement of registration may pose a barrier to accessing the public subsidy. Moreover we conjecture that ethnic characteristics may negatively influence access to the public subsidy; for instance, American studies found that Hispanic and immigrant children who are eligible for publicly available health insurance are more likely to remain uninsured than other program-eligible children [8,9]. A lack of insurance amongst program-eligible children has been attributed to parents' lack of knowledge, language barriers, immigration issues, miscommunication and perceived discrimination [3,8-11].

Given these findings, we sought to determine whether registration for the Fair PharmaCare program in British Columbia, Canada differed across areas differing in ethnic composition. British Columbia is an interesting case study to examine the effect of area-level ethnic composition because BC has one of the highest proportions of visible minorities of any Canadian province [12]. Approximately one in four (24.8%) of British Columbia's 4.2 million residents identifies as a visible minority. The most common ethnic identities reported include Chinese and South Asian (East Indian, Punjabi, Pakistani), accounting for 10% and 6.4% of British Columbia's total population, respectively.

### Conceptual Framework

**Figure 1:**
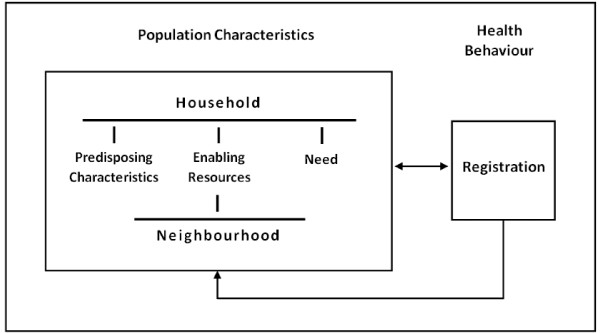
**Conceptual Model adapted from Phillips et al (1998)**.

In order to examine whether area-level ethnic composition influenced households' probability of registering for the Fair PharmaCare program in 2003, we used a modified version of the Andersen-Newman framework for health services utilization proposed by Philips et al [13,14] (see Figure 1). Similar to the original model, this version identifies three dimensions of population characteristics that act as predictors of health behaviour; namely: predisposing characteristics, enabling resources and need. Where the model differs, thereby making it more suitable for this study is the focus on and inclusion of "contextual" variables and the expansion beyond health services utilization as the outcome of interest.

Contextual variables measure the context in which utilization occurs and include environmental and provider related factors [14]. Environmental variables include health care delivery system characteristics, the external environment (e.g. the economic climate) and community-level enabling variables. Provider related factors refer to provider characteristics (e.g. physician gender, physician prescribing practices, etc) as well as patient factors that are influenced by providers (e.g. use of preventative screening).

Of particular interest for the purposes of this study are community-level enabling resources. In the framework proposed by Phillips et al, community-level enabling resources refer to "attributes of the community where individuals live that enables individuals to obtain services" [14]. Such resources include human and structural resources, such as the availability of physicians or hospitals in a community. Here we hypothesize that the collective cultural characteristics of residents within an area contribute to the community's social capital and thus are community-level enabling resources.

In addition to the inclusion of contextual variables, the model proposed by Phillips et al expands beyond health services utilization as the outcome of interest [14]. Instead, health behaviours are identified as the ultimate outcome of interest, with health services utilization and personal health choices included as subsets of health behaviours. This focus more appropriately represents the outcome of interest for this study, namely registration for Fair PharmaCare. Typically, health services utilization refers to the use of services that are offered by a health care provider (e.g. physician, nurse, etc). Health behaviours, on the other hand, more broadly refer to actions that impact health.

Finally, consistent with previous research examining household behaviours, we further adapt this framework to reflect the household as the unit of analysis, rather than an individual (see Figure 1) [15]. Registration for Fair PharmaCare does not occur at an individual-level; rather families or household members are required to register together. Consequently, we conjecture that the collective characteristics, resources and needs of household members influence registration for the program. Therefore, this study employs a conceptual model which examines how household-level predisposing characteristics, enabling resources and need, in conjunction with community-level enabling resources, influences registration for Fair PharmaCare.

## Methods

### Study Cohort

Our study cohort includes all 2003 British Columbia households who were eligible for coverage under the Fair PharmaCare program. The unit of analysis is the household because the program (and registration for it) operates at the household level. We excluded households with persons aged 65 years and older because they would have received public drug benefits as an age-entitlement prior to the introduction of Fair PharmaCare and were individually mailed registration packages prior to the implementation of the new program. We also excluded social assistance recipients, veterans, and other specific groups (e.g. registered Aboriginals) who received public drug benefits through programs other than Fair PharmaCare. Our focus on eligible households where persons were less than 65 years old allows us to explore registration rates among the population for which the Fair PharmaCare program was genuinely a new public drug benefit.

### Data Sources

Data compiled for this study included linked observations from a variety of patient-specific administrative health care databases and area-based measures that were custom tabulated for our research by Statistics Canada. Administrative health care databases included the British Columbia Linked Health Database (now contained within Population Data BC), which provided demographic, medical, and hospital records and BC PharmaNet, which provided pharmaceutical utilization records. Unless otherwise specified, our research data pertain to the year 2003, the year in which Fair PharmaCare was implemented. Area-based measures provided by Statistics Canada were derived from the Canadian Census (2001). Approvals were obtained from all relevant data stewards and the University of British Columbia (UBC) research ethics board.

### Data Analysis

To determine whether the probability of registration for the public drug plan varied across areas of differing ethnic composition, we conducted multivariate logistic regression analyses. The dependent variable was whether or not any member of a household had registered for Fair PharmaCare at any time during 2003. The selection of independent research variables was based on our conceptual framework. Univariate logistic regression analyses were also conducted to assess differences between registrants and non-registrants for each of the independent research variables.

### Research Variables

The primary independent variables of interest are measures summarizing immigration rates, concentrations of specific ethnicities, and overall ethnic diversity for the local area in which households live. These area-based measures reflect the aggregate characteristics of individuals by Census Dissemination Area. Census Dissemination Areas are the smallest level of aggregate data for which census profiles are made. British Columbia is divided into 7,463 Census Dissemination Areas containing between 50 and 2,000 residents in 2001 (mean = 585, median = 540). Owing to data privacy concerns, categorical indicators of local area characteristics were constructed such that specific Census Dissemination Areas could not be identified.

A categorical immigration variable was constructed to identify whether a household lived in an area with a relatively high proportion of recent immigrants (immigration within 5 years of the 2001 Census). Specifically, this variable identified whether a household lived in one of the top 5% of Census Dissemination Areas in terms of recent immigration (all such areas had recent immigration rates exceeding 18%). Categorical variables were also constructed to identify whether a household resided in an area with significant concentrations of a particular ethnic group. An area was considered to have "high" ethnic concentration if at least 30% of its residents identified with a particular ethnic group.

Based on Statistics Canada broad categorizations, the ethnic groupings used in this study were North American, British Isles, French, other European, Aboriginal, South Asian, Chinese, and other Asian. Note that, unlike the American census, which asks individuals to classify themselves into one of six racial categories (American Indian or Alaska Native; Asian; Black or African American; Native Hawaiian or Other Pacific Islander; White; and Other Race) and one of two ethnicities (Hispanic or Latino and Not Hispanic or Latino) [16], the Canadian census does not ask individuals to identify with a racial or ethnic category. Instead, the Canadian census asks individuals to identify their ethnic ancestry from a long, open-ended list of potential ethnic backgrounds [17]. Therefore, these non-mutually exclusive broad categorizations reflect the diversity of ethnic ancestries and cultures with which the Canadian population identifies. A minority of ethnic ancestries were not captured through these categorizations, as there were insufficient numbers of local areas in British Columbia with necessary ethnic concentrations to study registration rates for areas defined by these ethnicities (e.g. African or South American).

An overall ethnic homogeneity measure was computed using the Herfindahl index, which is the sum of the squared proportions of every ethnicity in an area [18]. The maximum value a Herfindahl index can take on is 1, in the hypothetical case that an area was comprised exclusively of a single ethnic group. We created a dichotomous variable that identified the 5% of Census Dissemination Areas that were most ethnically homogeneous based on this index. All of these areas had a Herfindahl index value exceeding 0.43, a level that could be achieved if an area had very high concentrations (> 60%) of at least one ethnicity or moderately high concentrations (> 40%) of two predominant ethnicities.

In addition to our primary independent variables of interest, based on our conceptual framework, variables that captured households' predisposing characteristics, enabling resources and need were included.

#### Predisposing factors

We adjusted for household size and composition due to potential relationships to health behaviors and perceived access to care [19-21]. Separate dichotomous variables identified households with at least one female adult; households with at least one child; and households headed by a single parent.

#### Enabling factors

Area-level income was assigned to households to provide a measure of neighborhood socioeconomic status. This measure was developed by Statistics Canada using 2002 tax filer data [22]. After calculating the average disposable income per person by postal code, postal codes were ranked by average income and then divided into 1000 income bands. The area-level income assigned to households corresponds to the income band associated with each household's postal code. We adjusted for local health system characteristics (e.g., level of urbanization, remoteness, and differences in supply of primary care providers) by including information about which of 89 Local Health Areas of the province households resided in. Local Health Areas are administrative regions of the province that differ in geography, population density and health system infrastructure. By including information about the Local Health Area in which households reside, regional fixed effects were accounted for.

We also adjusted for the likelihood that households had access to private drug coverage using information about employer-paid health benefits. This information was taken from administrative data that indicate whether any individual in a household worked for an organization that paid their public medical premiums on their behalf. Such arrangements are an (admittedly-imperfect) indicator of availability of employment-related extended health benefits, including private pharmaceutical coverage. In British Columbia, as of 2001, 47% of prescription drug expenditures are privately financed (either by out-of-pocket payment or private insurance) [23]. The balance of prescription drug expenditures is covered through provincial drug benefit programs (47%), federal drug benefit programs (5%) and the Workers Compensation Board (1%).

#### Need factors

Household-level health care needs were created by aggregating health status measures for individual household members. For each household member, all diagnostic codes were obtained from all administrative records of physician visits or hospital discharges during the year 2003 [24]. Patient comorbidities were identified from these diagnostic data using the 32 distinct morbidity clusters defined by the Aggregated Diagnostic Groups (ADGs) of the Adjusted Clinical Group system [25]. Households were categorized based on whether their members had diagnostic records indicating a total of zero, one, two, three, or four or more ADGs.

To gauge prospective needs for prescription drug coverage, we used total household drug costs in 2002. Categorical variables indicated whether a household had no prior drug expenditures, low prior drug expenditures (< CAD$150), medium prior drug expenditures (CAD$150 - CAD$500), high prior drug expenditures (CAD$500 - CAD$1,000), or catastrophic prior drug expenditures (over CAD$1,000). Prospective (2002) drug expenditures were used instead of concurrent (2003) drug expenditures to avoid model endogeneity stemming from the possibility that registration for public drug benefits affected the level of drug spending households could afford in the concurrent period.

## Results

Our study cohort included approximately 1.3 million households; excluded households (approximately 600 000) included households with members aged 65 or older, social assistance recipients, and/or members likely to receive prescription drug insurance through another publicly funded program. Across all households, almost 7% of the households lived in areas characterized by a high concentration of recent immigrants (see Table 1). A majority (≥ 70%) of the households lived in areas with high concentrations of individuals whose self-identified ethnicity falls into categories of British or other European (excluding French). Recall that area ethnic categorizations are non-mutually exclusive; almost 10% of areas identified as ethnically concentrated were characterized by more than one ethnic concentration (e.g. British, other European, and/or North American) (data not shown). Fewer households resided in areas characterized by high concentrations of persons self-identifying as Chinese (14.0% of all households), South Asian (4.7%), other Asian (1.8%), Aboriginal (0.77%), and French (0.16%). Only 2.5% of households in this study lived in areas that had no ethnic group accounting for at least 30% of the population. Similarly, only 1.8% of households in our cohort were located in very highly ethnically homogenous areas (as measured using the Herfindahl index).

**Table 1:** Descriptive statistics for non-senior households eligible for Fair PharmaCare during 2003

	% ( mean) of non-registrants	% (mean) of registrants	Total % (mean) of the population
Predisposing household characteristics			
Household size*			
One person	60.3	52.2	55.5
Two persons	16.8	22.5	20.2
Three or four persons	19.2	21.6	20.6
Five or more persons	3.7	3.7	3.7
Household composition			
At least one adult female*	58.3	74.0	67.6
At least one child*	38.1	26.3	25.8
Single-parent*	4.8	5.4	5.1

Enabling household characteristics			
Income (mean)	($34,581)	($34,149)	($34,326)
Private Insurance*			
Yes	39.2	48.3	44.6

Household needs			
Prescription drug expenditure (2002)*			
None ($0)	36.3	21.2	27.4
Low (< $150)	33.8	31.1	32.2
Medium ($150-500)	19.1	23.7	21.8
High ($500-1000)	6.9	11.3	9.5
Catastrophic (> $1000)	3.9	12.7	9.1
Total ADGs*			
Zero	23.2	10.5	15.7
One	12.4	9.2	10.5
Two	11.3	10.1	10.6
Three	9.8	10.0	9.9
Four or more	43.3	60.2	53.3

Local area characteristics			
Recent immigrant concentration*			
High	6.6	7.2	6.9
Ethnic concentration			
British Isles*	78.1	74.4	75.9
Other European*	71.7	68.9	70.0
North American*	32.7	31.0	31.7
Chinese*	12.3	15.1	14.0
South Asian*	4.0	5.2	4.7
Other Asian	1.8	1.9	1.8
Aboriginal*	0.95	0.64	0.77
French*	0.19	0.15	0.16
No ethnic concentration	2.4	2.5	2.5
Ethnic composition*			
Homogeneous	1.6	1.9	1.8

Households (N)	535,106	771,166	1,306,272

* Statistically significant (p < 0.05) difference between registered and non-registered.

With respect to Fair PharmaCare registration, approximately 60% of our study cohort registered for the program in 2003. Registered households differed from those who did not register in most household-level predisposing, enabling and need characteristics, but not in area-level income (see Table 1). Area-level ethnic characteristics also significantly differed across Fair PharmaCare registrants and non-registrants.

Table 2 reports adjusted odds ratios for the variables of interest. After controlling for household-level predisposing, enabling and need factors, measures of local area ethnic composition were significantly associated with likelihood of households' registration for the Fair PharmaCare program (p < 0.05). Adjusted odds of program registration among households living in areas of high rates of recent immigration were slightly lower than other areas (OR = 0.97, p < 0.05).

**Table 2:** Adjusted odds ratios of non-senior households' likelihood of registering for Fair PharmaCare during 2003

	Adjusted Odds Ratio	95% Confidence Interval
Predisposing household characteristics		
Household size		
One person	Ref	
Two persons	0.67	(0.67, 0.68)
Three or four persons	0.72	(0.71, 0.74)
Five or more persons	0.65	(0.63, 0.67)
Household composition		
Without a female adult	Ref	
With at least one adult female	1.74	(1.73, 1.76)
Without children	Ref	
With at least one child	0.71	(0.70, 0.72)
Not single-parent	Ref	
Single-parent	1.56	(1.53, 1.60)

Enabling household characteristics		
Private Insurance		
No	Ref	
Yes	1.18	(1.17, 1.19)

Household needs		
Prescription drug expenditure (2002)		
None ($0)	Ref	
Low (< $150)	1.21	(1.20, 1.23)
Medium ($150-500)	1.45	(1.43, 1.47)
High ($500-1000)	1.89	(1.86, 1.92)
Catastrophic (> $1000)	3.72	(3.66, 3.79)
Total ADGs		
Zero	Ref	
One	1.44	(1.42, 1.46)
Two	1.59	(1.56, 1.61)
Three	1.71	(1.68, 1.73)
Four or more	2.10	(2.07, 2.12)

Local area characteristics		
Recent immigrant concentration		
Average/low	Ref	
High	0.97	(0.95, 0.98)
Ethnic concentration		
British Isles	Ref	
Other European	0.99	(0.98, 1.00)
North American	0.99	(0.98, 1.00)
Chinese	1.21	(1.19, 1.23)
South Asian	1.19	(1.16, 1.22)
Other Asian	1.07	(1.04, 1.10)
Aboriginal	0.78	(0.74, 0.82)
French	0.92	(0.84, 1.01)
No ethnic concentration	1.07	(1.04, 1.10)
Ethnic composition/diversity		
Heterogeneous	Ref	
Homogeneous	1.12	(1.08, 1.15)

Adjusted odds of registration in areas with high concentrations of European, North American, and French were not significantly different than in areas with concentrations of British ethnicity (p < 0.05). Households located in areas with concentrations of Asian ethnicities had higher odds of program registration: Chinese (OR = 1.21, p < 0.05), South Asian (OR = 1.19), and other Asian (OR = 1.07). The only type of ethnic concentration shown to have a negative influence on the likelihood of households' registration was Aboriginal (OR = 0.77). Households residing in areas in which no ethnic group accounted for greater than 30% of the local population were more likely to register for the program than areas with concentrations of British ethnicity (OR = 1.07). At the other end of the spectrum, households living in highly ethnically homogeneous areas were more likely to register for the program than other households (OR = 1.12).

### Predisposing factors

Most household characteristics modeled in this study bore a statistically significant relationship to the likelihood of registering for the Fair PharmaCare program. Single individuals were more likely to register than families of any other size. Households with at least one female adult were more likely to register than households without a female adult (OR = 1.74). While households with at least one child were less likely to register for Fair PharmaCare than households without children (OR = 0.71), single-parent households were more likely to register than other household types (OR = 1.56).

### Enabling factors

Households likely to be covered by employment-related private drug insurance were more likely to have registered than other households (OR = 1.18). The Local Health Area variables included to capture fixed effects of regional context and health system resources had significant effects on the adjusted odds of registering for the Fair PharmaCare program; the most notable regional effect was that households situated in northern and remote regions were less likely to register for the Fair PharmaCare program (results not reported in Table 2).

### Needs

Households' likelihood of registration was correlated with measures of health needs. The adjusted odds of registering for the Fair PharmaCare program was significantly higher across the progressively higher categories of prescription drug costs for 2002 (p < 0.05). Similarly, the odds of registration were positively associated with total number of household co-morbidities during 2003.

## Discussion

Our results suggest that the likelihood of registration for a new public drug benefit program in British Columbia was affected by area-level ethnicity. In particular, higher area-level concentration of certain ethnic groups was associated with increased likelihood of program registration even after adjusting for household-level factors such as health care needs, household composition, socioeconomic status, and likelihood of access to private insurance. Because our models included the total cost of prescription drugs used by each household during the year prior to the new program (year 2002), the effect of area-level ethnicity captured here is likely to be independent of ethnic variations in the use of prescribed medicines themselves.

Our findings are consistent with US studies showing that individuals report better access to care when the predominant ethnic group in their county of residence corresponds with their own ethnic background [26]. However, given that households living in areas with high concentrations of Chinese or South Asian ethnic groups had higher rates of registration, we investigated the communication strategies employed by the government of British Columbia to promote the Fair PharmaCare program and to encourage registration. We learned that, due to the diverse population residing in British Columbia, the government took steps to alleviate potential barriers to access. In addition to promoting the program to the public through English language media, promotional advertisements were disseminated in Chinese and Punjabi: the two most prominent ethnic languages spoken amongst residents. Chinese and Punjabi residents were also able to obtain translated registration forms and translational services through the program's registration call-centre.

In contrast to our findings related to households residing in areas of high Chinese or South Asian ethnic concentrations, we noted a relatively lower likelihood of registration for households residing in areas of high Aboriginal ethnic concentration. It is important to note that neither the BC Fair PharmaCare program nor our research data pertain to registered Aboriginal households because their health care (including drug benefits) is under federal jurisdiction in Canada. Nevertheless, not all individuals who identify as Aboriginal are registered and not all households in areas characterized by high Aboriginal concentration consist of individuals who identify as Aboriginal. Lower rates of Fair PharmaCare participation among eligible persons living in areas of significant Aboriginal population likely reflect important local cultural, socioeconomic, and policy contexts that deserve further study. To do so will require addressing significant privacy considerations and administrative data shortcomings regarding Aboriginal identification [27].

Several interesting household-level findings warrant further discussion. The first relates to our finding that households more likely to be covered by private drug insurance are more likely to register for the public drug benefit program. This finding can be explained by policies introduced by private drug companies in response to the creation of the income-based Fair PharmaCare program, which does not discriminate between households with and without private drug benefits. Private drug companies now require that their clients in British Columbia enroll in the Fair PharmaCare program to be eligible for their private drug benefits. Such policies protect private drug companies, as households with high drug expenditures relative to their incomes may qualify for public drug benefits, which in turn would shift the cost burden from the private sector to the public sector.

Another potentially counter intuitive finding related to the impact of both household size and the presence of children: larger households and households with at least one child were less likely to have registered for the public drug benefit program. While this finding may seem surprising, it is consistent with past research. Chen and Escarce (2006) examined the impact of family structure on children's use of ambulatory care and prescription medicines in the USA [19]. Similar to our findings, their study found children's use of ambulatory care and prescription medicines decreased as family size increased. A plausible explanation for these findings is the notion of finite parental resources: as family size increases, parental resources are increasingly divided and strained. It has been suggested that the additive needs of each additional family member on the head(s) of a household results in decreased use of health services for all family members. With respect to registration for Fair PharmaCare, it is plausible that larger families were less likely to register for the program due to time restraints on the household head(s). However, further research is needed to understand whether members of larger families are less aware of public programs, are misinformed regarding potential benefits or are too busy to engage in the registration process.

In accordance with our findings related to family size, families with at least one child were 29% less likely to have registered for Fair PharmaCare than families lacking children. Again, considering past research, studies have shown that enrolment for public health insurance in the USA differs between single parent families and two parent families, suggesting that the resources of the parent(s) (or lack thereof) is the mediating factor influencing enrolment - not the presence of children [28]. Indeed our findings support this notion, as single parent families were 56% more likely to register for Fair PharmaCare than any other family type. We hypothesize that families with children have more demands on their time than families lacking children, limiting their opportunities to register. In contrast, we hypothesize that single parent families face greater financial burdens, and thus had greater incentives to register for the program. Additional research is needed to compare health behaviours between families with varying family compositions, including single and two-parent families as well as families without children. To date, studies have focused largely on families with children and differing parental structures [28-30].

The primary limitation to this analysis relates to our data permissions, which prohibited the identification of dissemination areas by its area-level ethnic characteristics. This restriction resulted in an inability to include some of the variables we thought belonged in our model. For example, we were unable to simultaneously include total and recent immigration rates in the analysis, since these measures created unique area-level ethnic profiles that could have been used to identify specific dissemination areas. Recent immigration was selected because it added more unique 'information' over and above our other area-based ethnicity variables. Furthermore, the conditions of our data permissions prohibited the use of multi-level statistical analyses.

## Conclusion

Our study contributes to the literature by demonstrating the utility of linking large administrative databases that lack ethnic data, with census data. Moreover, changes to our model (results not shown) did not alter our study's findings, suggesting that our study's results are robust. Specifically, this study identified ethnic variation in registration for a new public drug benefit program in British Columbia. We did not find evidence that area-level ethnic concentration created access barriers to the Fair PharmaCare program, relative to areas with higher concentrations of households that identified with a British ethnicity. Instead, households with the highest likelihood of registration for Fair PharmaCare resided in areas with higher concentrations of Chinese or South Asians. To further understand why ethnic minorities, particularly Chinese and South Asians achieved higher registration rates, further research is needed. In particular, research that examines the communications strategies employed by the government of British Columbia may provide insight into how to improve uptake of public programs. Given the experiences of ethnic minority communities in other jurisdictions, as well as the Aboriginal and recent immigrant communities in British Columbia, this research may inform governments struggling with underserved populations.

## Competing interests

The authors declare that they have no competing interests.

## Authors' contributions

This manuscript represents the thesis work of VWL. VWL participated in the conceptualization of the research objectives, study design and acquisition of data, carried out data analyses, and drafted the manuscript. As VWL's thesis supervisor, SM participated in the conceptualization of the research objectives and study design, facilitated the acquisition of data and assisted to draft the manuscript. STW supported the conceptualization of the study design and research objectives, facilitated the acquisition of data and assisted to draft the manuscript. GEH contributed to the acquisition of data and development of databases; assisted with data analyses and the interpretation of results. CB supported the conceptualization of the study design and research objectives. All authors read and approved the final manuscript.

## Authors' Information

VWL completed her Master's of Science at the University of British Columbia in the Department of Health Care and Epidemiology (now the School of Population and Public Health) in August 2008. This manuscript is based on her thesis work, which was completed at the UBC Centre for Health Services and Policy Research (CHSPR). She now works as a Policy Advisor at the Council of Academic Hospitals of Ontario (CAHO).

## Pre-publication history

The pre-publication history for this paper can be accessed here:

http://www.biomedcentral.com/1472-6963/10/171/prepub
